# Inhibitory effect of herbal medicines and their trapping abilities against methylglyoxal-derived advanced glycation end-products

**DOI:** 10.1186/s12906-015-0897-8

**Published:** 2015-10-31

**Authors:** Weerachat Sompong, Sirichai Adisakwattana

**Affiliations:** Department of Nutrition and Dietetics, Faculty of Allied Health Sciences, Chulalongkorn University, Bangkok, 10330 Thailand

**Keywords:** Advanced glycation end-products, Methylglyoxal, Herbal medicines

## Abstract

**Background:**

Methylglyoxal (MG) is one of the most reactive glycating agents, which result the formation of advanced glycation end-products (AGEs) that have been implicated in the progression of age-related diseases. Inhibition of MG-induced AGE formation is the imperative approach for alleviating diabetic complications. The objective of this study was to investigate the MG-trapping abilities of herbal medicines and their inhibitory activities on the formation of MG-derived AGEs.

**Methods:**

The aqueous extract of herbal medicines was measured for the content of total phenolic compounds and the antioxidant activity by Folin-Ciocalteu assay and the 1,1-diphenyl 2-picrylhydrazyl (DPPH) radical scavenging activity, respectively. The extracts were investigated the MG-trapping ability by high performance liquid chromatography (HPLC). The extracts were incubated with BSA and MG at 37 °C for 1 day. The formation of MG-derived AGEs was measured.

**Results:**

Total phenolic compounds of eleven herbal medicines showed marked variations, ranging from 12.16 to 272.36 mg gallic acid equivalents/g extract. All extracts (1 mg/mL) markedly exhibited the DPPH radical scavenging activity (0.31–73.52 %) and the MG-trapping abilities (13.97–58.97 %). In addition, they also inhibited the formation of MG-derived AGEs by 4.01–79.98 %. The results demonstrated that *Rhinacanthus nasutus, Syzygium aromaticum*, and *Phyllanthus amarus* were the potent inhibitors against the formation of MG-derived AGEs. The positive correlations between the contents of phenolics and % MG trapping (*r* = 0.912, *p* < 0.01) and % inhibition of MG-derived AGEs (*r* = 0.716, *p* < 0.01) were observed in the study. Furthermore, there was a moderate positive correlation between % MG trapping and % inhibition of MG-derived AGEs (*r* =0.584, *p* < 0.01).

**Conclusions:**

*Rhinacanthus nasutus, Syzygium aromaticum*, and *Phyllanthus amarus* could reduce the formation of MG-derived AGEs through their MG-trapping abilities. These findings are relevant for focusing on potential herbal medicines to prevent or ameliorate AGE-mediated diabetic complications.

## Background

Diabetes mellitus is a chronic progressive metabolic disorder characterized by hyperglycemia, dyslipidemia, and protein metabolisms. Chronic hyperglycemia of diabetes increases a risk of relatively developing microvascular and macrovascular complications associated with reduced quality of life and increased risk of mortality and morbidity [1]. Advanced glycation-end products (AGEs) are the results of protein glycation occurring by the reaction between proteins and reducing sugars through a complex process including further rearrangement, oxidation, dehydration, and polymerization [2]. AGEs are possible causal factors for development of multiple features of diabetes and its complications such as cardiovascular diseases, neuropathy, retinopathy, and nephropathy [2]. Methylglyoxal (MG), a reactive α-dicarbonyl compound, is an important precursor of early glycation. It has been shown that MG is produced from spontaneous phosphate elimination of glycolytic pathway [3]. Other sources of MG are in sugar-containing foods and beverages such as bread, coffee, honey, wine, and beer [4]. Several studies have shown that the higher levels of MG were observed in diabetic patients [5, 6]. Evidences support that MG is the most potent glycating agent among the reactive compounds [7, 8]. In addition, the glycation reaction of amino acid with MG also generates reactive oxygen species (ROS) associated with the induction of oxidation-dependent damage to protein and DNA [9, 10]. Aminoguanidine (AG), a well-known antiglycating agent inhibits the formation of AGEs. However, AG has been terminated due to serious side effects such as myocardial infarction, congestive heart failure, atrial fibrillation, anemia, and gastrointestinal disturbance [11]. Studies on AGEs inhibitors from naturally occurring compounds have emerged as a new strategy to reduce AGEs-associated diseases [12].

Herbal medicines have been suggested as an alternative source of potentially useful anti-diabetes and antiglycation. In China and other Asian countries, herbal medicines have been widely used for treatment and prevention of many diseases for a long time. Several pharmacological activities of herbal medicines have been shown including antihyperlipidemic [13], anti-diabetic [14], antiulcer [15], and anti-inflammatory activities [16]. Findings from several published literature have reported antidiabetic activity of herbal medicines related to delay carbohydrate digestion and absorption [17-18]. Moreover, numerous medical herbal medicines and dietary plants have been reported to inhibit fructose-induced protein glycation such as sweetleaf, penneywort, gingko, senna, and safflower [19]. It was found that pennywort extract had the highest percentage of glycation inhibition among the extracts. Traditional Chinese medicines have been investigated for the inhibition of AGE formation using fructose and glucose models [20]. Among herbal medicines used in the study, *Flos Sophorae Immaturus* and *Radix Scutellariae* had the highest effective on inhibiting the formation of AGEs [20]. As listed in Table 1, they have been commonly used in the Ayurvedic system of Thai traditional medicine to treat various diseases. Interestingly, they have been described in the scientific literature as having antidiabetic activity and their mechanisms [21-28]. However, there were limited data available demonstrating the preventive mechanisms of herbal medicine on diabetes and its complications related to the inhibition of formation of MG-derived AGEs. In this regard, the aim of present study was to investigate the MG-trapping abilities of herbal medicines using high performance liquid chromatography (HPLC). In addition, the inhibitory effect of herbal medicines on the formation of MG-derived AGEs was also investigated. Moreover, the antioxidant activity and total phenolic content were examined in order to evaluate their possible relationships with the MG-trapping abilities and the formation of MG-derived AGEs.

**Table 1:** The list of plants was used of this study

Plant samples		
Scientific name	Family	Used part
*Rhinacanthus nasutus*	Acanthaceae	Leaves
*Cissus quadrangularis*	Vitaceae	Aerial parts
*Syzygium aromaticum*	Myrtaceae	Buds
*Acanthus ebracteatus*	Acanthaceae	Leaves
*Thunbergia laurifolia*	Acanthaceae	Leaves
*Phyllanthus amarus*	Euphorbiaceae	Aerial parts
*Cassia alata*	Leguminosae	Leaves
*Pluchea indica*	Asteraceae	Aerial parts
*Schefflera leucantha*	Araliaceae	Leaves
*Cryptolepis buchanani*	Asclepiadaceae	Aerial parts
*Derris scandens*	Fabaceae	Aerial parts

**Table 2:** Total phenolic content, % MG trapping, % DPPH radical scavenging activity, and % inhibition of MG-derived AGEs of extracts (1 mg/mL)

Plant samples	Total phenolic content (mg/g extract)	% MG trapping	% DPPH radical scavenging activity	% Inhibition of MG-derived AGEs
*Rhinacanthus nasutus*	90.98 ± 0.61	20.87 ± 0.92	11.79 ± 2.59	79.98 ± 5.58
*Cissus quadrangularis*	12.16 ± 0.47	13.97 ± 1.36	0.31 ± 0.03	4.60 ± 1.85
*Syzygium aromaticum*	272.36 ± 4.50	58.97 ± 3.07	73.52 ± 0.62	60.73 ± 0.74
*Acanthus ebracteatus*	25.96 ± 0.28	15.70 ± 3.34	4.44 ± 1.00	N.I.
*Thunbergia laurifolia*	85.64 ± 0.64	30.88 ± 0.51	19.37 ± 2.81	N.I.
*Phyllanthus amarus*	201.72 ± 1.89	55.91 ± 2.76	54.08 ± 1.00	63.70 ± 2.05
*Cassia alata*	88.66 ± 0.42	32.69 ± 1.23	11.33 ± 2.29	30.08 ± 0.97
*Pluchea indica*	60.55 ± 0.30	20.77 ± 2.58	12.91 ± 1.63	N.I.
*Schefflera leucantha*	35.25 ± 0.94	25.35 ± 0.96	2.87 ± 1.51	4.01 ± 0.86
*Cryptolepis buchanani*	84.19 ± 0.47	23.53 ± 2.56	20.38 ± 1.46	4.03 ± 0.43
*Derris scandens*	106.75 ± 0.62	42.18 ± 1.89	2.13 ± 0.9	26.23 ± 0.68
Aminoguanidine	N.D.	99.58 ± 0.21	N.D.	79.54 ± 0.79
Gallic acid	N.D.	N.D.	87.85 ± 2.28	N.D.

Results are represented as mean ± SEM (*n* = 3). *N.D.* = not determined *N.I.* = No inhibition

## Methods

### Chemicals

Methylglyoxal (40 % in water), bovine serum albumin (BSA, fraction V), aminoguanidine, 2-methylquinoxaline, 5-methylquinoxaline, *o*-phenylenediamine, 2,2-diphenyl-1-picrylhydrazyl (DPPH), Folin-Ciocalteu’s phenol reagent, and gallic acid were purchased from Sigma-Aldrich (St. Louis, MO, USA). All other chemicals used were of analytical grade.

### Plant material

The plants were purchased from a specific herbal drugstore, Bangkok, Thailand. The plant has been authenticated at the Princess Sirindhorn Plant Herbarium, Plant Varieties Protection Division, Department of Agriculture, Thailand. The plants (20 g) were boiled in distilled water (800 mL) for 3 h at 95 °C. After boiling, the plant residue was filtered through Whatman No. 1 filter paper. Thereafter, the extraction was lyophilized with a freeze drier. The lyophilized powder was stored at 4 °C in a dark bottle until analysis. The powder of extract was resuspended in distilled water before experiments.

### Determination of total phenolic content

The total phenolic content of extracts was done according to a previous method [19]. The extract was freshly dissolved in distilled water before use. Briefly, 10 μL of sample solution (1 mg/mL) was mixed with 100 μL of Folin-Ciocalteu’s reagent (10-fold dilution in distilled water before use). After incubation for 5 min at room temperature, then 80 μL of 1 M sodium carbonate solution was added, and the mixture was allowed to stand for 60 min at room temperature before the absorbance of the reaction mixture was measured at 760 nm. The concentration of gallic acid (0–1,000 μg/mL) was used for a standard curve. The total phenolic contents were calculated using a standard curve and expressed as milligram of gallic acid equivalents per gram of extract.

### DPPH radical scavenging activity

DPPH (1,1-diphenyl 2-picrylhydrazyl) radical scavenging activity was measured according to the previous method [29]. Briefly, the extract was added with 0.2 mM DPPH as the free radical source and incubated for 30 min at room temperature. The decrease in the solution absorbance was measured at 515 nm. The percentage of DPPH radical scavenging activity was calculated according to the equation below:

$$ \%\;\mathrm{DPPH}\;\mathrm{radical}\;\mathrm{scavenging}\;\mathrm{activity} = \left[1\hbox{-}\ \left({\mathrm{Abs}}_{\mathrm{sample}}/{\mathrm{Abs}}_{\mathrm{control}}\right)\right]\times 100 $$

Gallic acid was used as a positive control for this study.

### Screening of the methylglyoxal-trapping ability by high performance liquid chromatography (HPLC)

The MG-trapping ability of extracts was done according to a previously published method with minor modifications [30]. The mixture of MG (1 mM) with the extract (1 mg/mL) or aminoguanidine (1 mg/mL) in phosphate buffer solution (pH 7.4) at 37 °C was incubated for 1 day. Quantification of methylglyoxal (MG) was based on the determination of its derivative compound, 2-methylquinoxaline (2-MQ) using HPLC with 5-methylquinoxaline (5-MQ) as the internal standard. The solution containing 20 mM *o*-phenylenediamine (100 μL) and of 5 mM 5-MQ (100 μL) was added into the sample vials immediately after incubation. MG derivatization took place at the room temperature. After 30 min, the samples were filtered and ready for HPLC analysis. The remaining MG in the samples was quantified using HPLC (Shimadzu Corp., Kyoto, Japan) equipped with a LC-10 AD pump, SPD-10A UV–vis detector and LC-Solution software. A C_18_ (Inertsil ODS 3 V) column (250 × 4.6 mm i.d.; 5-μm particle size) was used for 2-MQ analysis. The column temperature was maintained at room temperature. The mobile phase for the HPLC system consisted of HPLC grade water (solvent A) and methanol (solvent B) with a constant flow rate set at 1.2 mL/min. In brief, aliquots of 10 μL were subjected to HPLC analysis. An isocratic program was performed with 70 % solvent B and 10-min running time per sample. The 2- and 5-MQ was monitored at 315 nm. Peak integrality ratios of 2-MQ to 5-MQ were used for quantitative analysis. The amount of MG was calculated by using the standard curve of 2-MQ/5-MQ. The percentage of MG trapping was calculated using the equation below:

$$ \%\;\mathrm{M}\mathrm{G}\;\mathrm{trapping}=\mathrm{Amount}\;\mathrm{of}\;\mathrm{M}\mathrm{G}\;\mathrm{in}\;\mathrm{control}\;\hbox{--}\;\mathrm{M}\mathrm{G}\;\mathrm{in}\;\mathrm{extract}/\mathrm{Amount}\;\mathrm{of}\;\mathrm{M}\mathrm{G}\;\mathrm{in}\;\mathrm{control}\times 100 $$

### The formation of methylglyoxal-derived AGEs

The formation of MG-derived AGEs was prepared according to the previous method with minor modifications [31]. 0.5 mL of BSA (20 mg/mL) was incubated with 0.4 mL of 2.5 mM MG and 0.1 mL of extract or AG (10 mg/mL) as positive control in 0.1 M phosphate buffer saline (PBS), pH 7.4 at 37 ° C for 1 day. The fluorescent intensity was measured to assess MG-derived AGE formation by using a spectrofluorometer Synergy HT (Biotek Instruments, Winooski, VT, USA) at the excitation wavelength of 355 nm and emission wavelength of 460 nm, respectively. The percentage inhibition of MG-derived AGE formation was calculated as following formula below:

$$ \mathrm{Inhibition}\;\mathrm{of}\;\mathrm{M}\mathrm{G}\hbox{-} \mathrm{derived}\;\mathrm{AGEs}\;\left(\%\right)=\left[\left(\left(\mathrm{F}\mathrm{C}\hbox{-}\ \mathrm{F}\mathrm{C}\mathrm{B}\right)\;\hbox{-}\;\left(\mathrm{F}\mathrm{S}\hbox{-}\ \mathrm{F}\mathrm{S}\mathrm{B}\right)\right)/\left(\mathrm{F}\mathrm{C}\hbox{-}\ \mathrm{F}\mathrm{C}\mathrm{B}\right)\right]\times 100 $$

Where FC and FCB were the fluorescent intensity of control with MG and blank of control without MG, FS and FSB were the fluorescent intensity of sample with MG and blank of sample without MG.

### Statistical analysis

Values were expressed as mean ± standard error of the mean (SEM) of three replicate determinations. Pearson’s correlation analysis was used to determine the correlation between total phenolic, % DPPH radical scavenging activity, % MG trapping, and % inhibition of MG-derived AGEs. A value of *P* < 0.01 was considered to be statistically significant.

## Results

### Total phenolic content

The total phenolic compounds of the extracts are presented in Table 2. Total phonolic compounds of eleven herbal medicines showed marked variations, ranging from 22.95 to 239.58 mg gallic acid equivalent/g extract. The highest and lowest amount of total phenolic compound was obtained from *Syzygium aromaticum* and *Cissus quadrangularis*, respectively.

### DPPH radical scavenging activity

The DPPH radical scavenging activity of eleven herbal medicines is presented in Table 2. The results demonstrated that all extracts (1 mg/mL) had the ability to scavenge free radicals by 0.31-73.52 %. It was found that the highest percentage of DPPH radical scavenging activity was elicited by *Syzygium aromaticum*. It is observed that *Cissus quadrangularis* had the lowest perecentage of DPPH radical scavenging activity in comparison with other herbal medicines.

### Methylglyoxal-trapping capacity

**Fig. 1:**
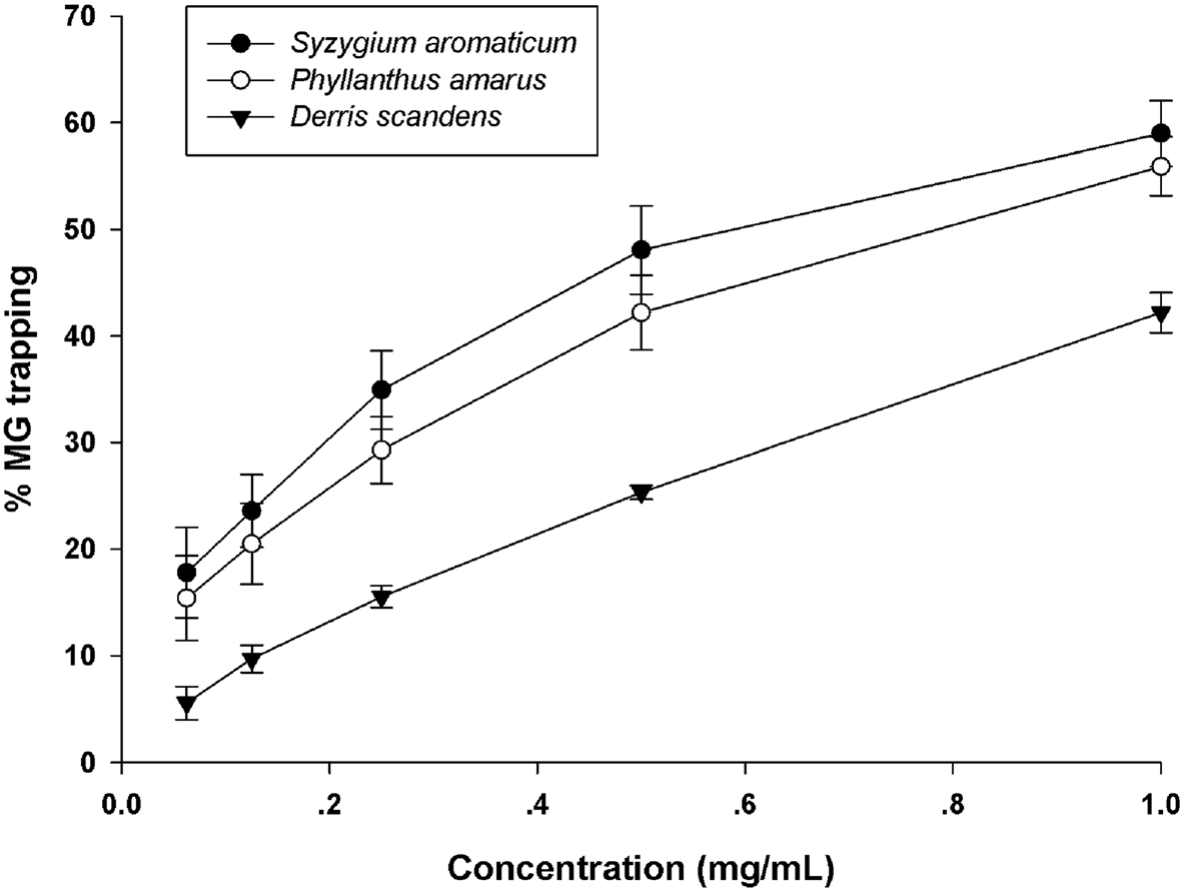
Concentration-dependent results for MG-trapping abilities of *Syzygium aromaticum, Phyllanthus amarus, and Derris scandens*. Results are expressed as mean ± SEM for *n* = 3

Table 2 shows the MG-trapping capacity of herbal medicines. An evaluation of direct MG-trapping ability was carried out in order to investigate whether herbal medicines could directly scavenge MG. At the concentration of 1 mg/ mL, *Syzygium aromaticum* was the most effective MG-trapping ability, whereas *Cissus quadrangularis* had the lowest potent MG-trapping ability among those of extracts. However, eleven herbal medicines were less potent than AG when compared at the same concentration. Based on the screening results, three herbal medicines with the potent MG-trapping abilities (*Syzygium aromaticum*, *Phyllanthus amarus,* and *Derris scandens*) were selected for further investigation (Fig. 1). The results demonstrated that *Syzygium aromaticum*, *Phyllanthus amarus,* and *Derris scandens* (0.0625-1 mg/mL) directly trap MG in a concentration-dependent manner (5.55-58.97 %).

### The formation of MG-derived AGEs

**Fig. 2:**
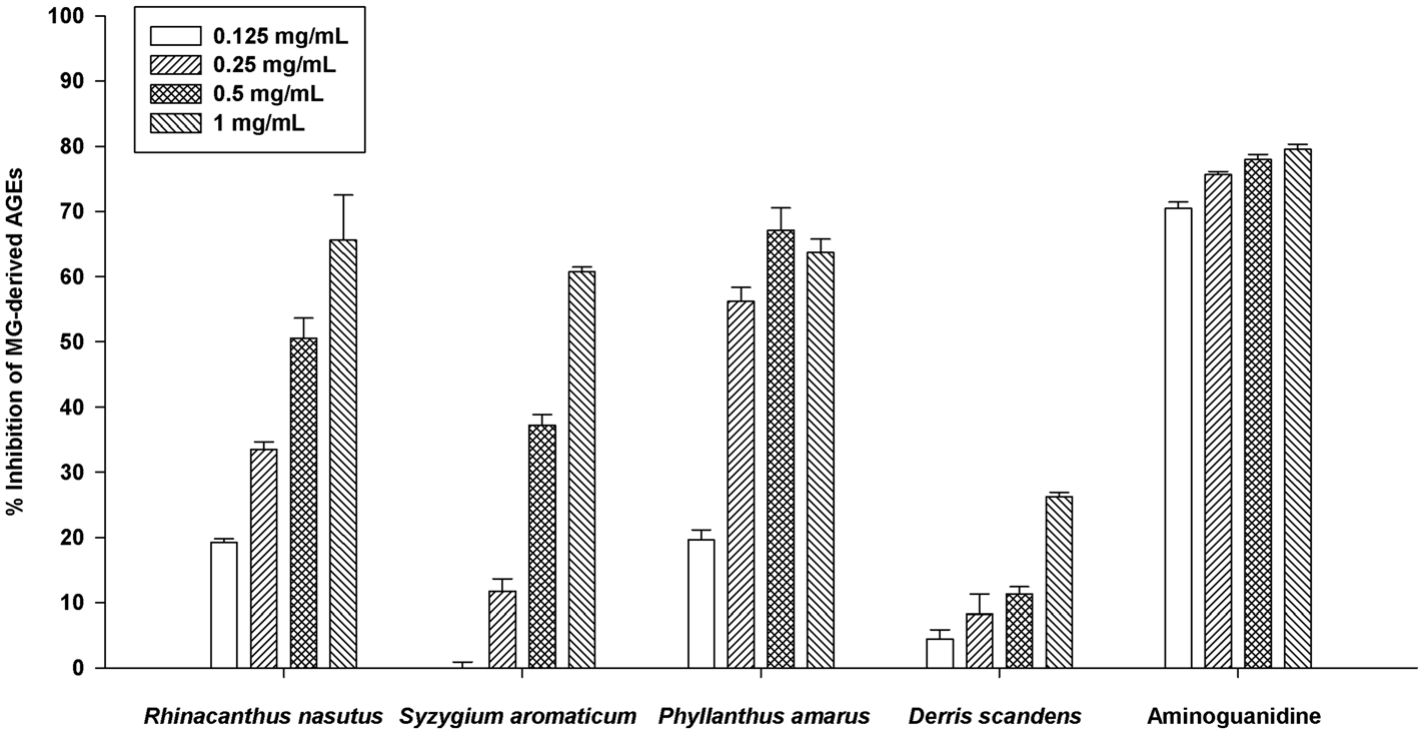
The percentage inhibition of *Rhinacanthus nasutus, Syzygium aromaticum, Phyllanthus amarus, Derris scandens, and aminoguanidine* (0.125-1 mg/mL) on the formation of MG-derived AGEs in BSA. Results are expressed as mean ± SEM for *n* = 3

According to the above-mentioned results (% inhibition of MG-derived AGEs and the MG-trapping ability), various concentrations of four herbal medicines were designed for incubation with MG and BSA (Fig. 2). The various concentrations of the extracts (0.125-1 mg/mL) markedly inhibited the formation of MG-derived AGEs in BSA. The percentage inhibition of *Rhinacanthus nasutus*, *Syzygium aromaticum*, *Phyllanthus amarus,* and *Derris scandens* was 33.54–79.98 %, 19.24–65.58 %, 19.62–67.13 %, and 4.46–26.63 %, respectively.

### Pearson’s correlation coefficients

The Pearson’s correlation coefficients between the variables are presented in Table 3. There were strong positive significant correlations between the contents of phenolic compounds and % MG trapping (*r* = 0.912, *p* < 0.01) and % inhibition of MG-derived AGEs (*r* = 0.716, *p* < 0.01). Furthermore, there was a moderate positive correlation between % MG trapping and % DPPH radical scavenging activity (*r* = 0.534, *p* < 0.01) and % inhibition of MG-derived AGEs (*r* = 0.584, *p* < 0.01). In contrast, % DPPH radical scavenging activity had no correlation with % inhibition of MG-derived AGEs.

**Table 3:** Pearson correlation analyses of total phenolic content, % MG trapping, % DPPH radical scavenging activity, and % inhibition of MG-derived AGEs of extracts (1 mg/mL)

	Total phenolic content	% MG trapping	% DPPH radical scavenging activity	% Inhibition of MG-derived AGEs
Total phenolic content	-	0.912^*^	0.531^*^	0.716^*^
% MG trapping	-	-	0.534^*^	0.584^*^
% DPPH radical scavenging activity	-	-	-	0.290

DPPH, 1,1-diphenyl-2-picrylhydrazyl radical-scavenging activity

^*^Correlation is significant at *P* < 0.01

## Discussion

MG is one of the reactive carbonyl species that are able to protein glycation resulting in the formation of advanced glycation end products (AGEs). The reaction of MG with lysine residues occurs irreversibly to form N^ε^-(1-carboxyethyl)lysine (CEL), MG-derived lysine dimer (MOLD). In particular, protein glycation by MG mainly reacts with arginine residues and mainly forms methylglyoxal-derived hydroimidazolone with a minor formation of argpyrimidine [32]. Methylglyoxal-derived hydroimidazolone-1 (MG-H1) is the most abundant MG-derived AGE in the human plasma, which contributes to various disease states such as diabetes [33], cancer [34], and cardiovascular diseases [35] by interacting with the receptor for advanced glycation end-products (RAGE) [32]. The reduction of MG-derived AGEs can be an effective strategy for prevention of diabetic complications. The direct trapping of MG is known as one of the mechanisms for the inhibition of MG-derived AGE formation [36]. Many fruits and vegetables have been recently evaluated their MG-trapping abilities [36]. The seed extract of apricot and peach had the highest ability to trap MG associated with the formation of MG-derived AGEs. Sugar-free extracts of berries (blueberry, strawberry, cranberry, raspberry, and blackberry) could scavenge MG correlated with the inhibition of protein glycation [37]. In addition, *Houttuynia cordata*, a native perennial herbaceous plant from the Saururaceae family, inhibited the formation of AGEs through a direct trapping MG [38]. Recent data indicate that some phytochemicals are able to react with MG. For instance, procyanidins from cinnamon were shown to scavenge MG [39]. In addition, resveratrol can form adducts when incubation with MG [40]. Two major anthocyanins from the blackcurrant extract, delphinidin-3-rutinoside (D3R) and cyanidin-3-rutinoside (C3R) had the MG-trapping ability by forming mono-MG adduct. Yoon et al. reported that vicinyl dihdroxyl groups of quercitrin and rutin are the active site for direct trapping of MG by forming mono- or di-MG adducts [38]. Flavonoids are ubiquitous in nature and many of which occur in herbal medicines. Researchers have documented the structure-activity relationships of flavonoids in scavenging MG. It has been shown that a ring of epigallocatechin gallate plays a vital role in trapping of reactive dicarbonyl species [41]. In addition, the presence of more hydroxyl groups on the phenyl ring result in the stronger the scavenging activity [42].

In the present study, it has been demonstrated that aqueous extracts of herbal medicine possess the MG-trapping ability and the inhibition of MG-derived AGEs. Previous studies have found the correlations between total phenolic compounds and the inhibitory effect on AGE formation of different extracts [39, 43]. Povichit et al. described negative correlation that medicinal plants exhibited high antiglycation activity while they had low phenolic content [44]. The present findings indicate that total phenolic compounds strongly correlate with the MG-trapping abilities of the extract, whereas they demonstrate moderate association with the inhibition of MG-derived AGEs. In addition, the MG-trapping abilities of the extracts were found to correlate with the inhibition of MG-derived AGEs in our experiments. These results were in agreement with a previous report regarding the correlation of MG-trapping ability and the formation of MG-derived AGEs [36]. Our results showed that no relationships between DPPH radical scavenging activity and the formation of MG-derived AGEs were found in the present study. Since MG induces the formation of AGEs through multiple steps, it is possible that free radical scavenging activity of extracts may not serve as a major mechanism against the formation of MG-derived AGEs.

The formation of AGEs generally includes three stages: early, intermediate, and late. At the early stage of glycation, protein reacts with reducing sugars resulting in the formation of Schiff base followed by rearrangement to an Amadori product [45]. Consequently, reactive dicarbonyls, particularly 3-deoxyglucosone and methylglyoxal are generated from autoxidation of glucose and the degradation of Amadori products, referred to as the intermediate stage of glycation [45]. In the late stage of glycation, irreversible products called AGEs are formed through direct degradation of Amadori products or Schiff bases, protein modification by dicarbonyl compounds and reactions between Amadori products and AGE precursors. According to our findings, *Syzygium aromaticum*, inhibited the formation of MG-derived AGEs. *Syzygium aromaticum*, the most widely cultivated spices in many tropical countries, has been used in traditional medicine since ancient times to treat respiratory and digestive ailments. Our previous results revealed that the extract of *Syzygium aromaticum* was capable to inhibit fructose-induced protein glycation [46]. The extract also reduced oxidation-induced protein damage concomitant with decreasing protein carbonyl formation and depletion of protein thiol group. The findings indicate that the extract prevented fructose-induced formation of AGEs in BSA at the initial stage of glycation resulting in reduced conversion of the initial glycated product to AGEs. In the current study, MG-induced formation of AGEs was also attenuated by *Syzygium aromaticum* at the intermediate stage of glycation. These findings, taken together, indicate that *Syzygium aromaticum* inhibit protein glycation both the initial and intermediate stages, thus leading to inhibition of the formation of AGEs in the late stage. *Phyllanthus amarus* belongs to the Euphorbiaceae family, which has been used to treat problems related to the genitourinary tracts [47]. Recent studies have also revealed antidiabetic activity of this extract [26, 48]. It is notable that *Phyllanthus amarus* exhibit considerable α-amylase inhibitory activities, which may suppress postprandial glucose [27]. *Rhinacanthus nasutus* (Linn) is a flowering plant that belongs to the Acanthaceae family. This plant has been used in traditional medicine for treatment of skin diseases [49]. It has been recently reported that *Rhinacanthus nasutus* improves the levels of carbohydrate and glycogen, and the liver markers in streptozotocin-induced diabetic rats [28]. The present findings demonstrate the antiglycation mechanism of *Phyllanthus amarus* and *Rhinacanthus nasutus* through its ability to trap MG leading to the inhibition of MG-derived AGE formation. Hence, the herbal medicines might prevent and delay the progression of diabetic complications through these mechanisms. Wu et al. proposed other mechanisms of antiglycation including particularly for inhibiting the formation of late-stage Amadori products, breaking the cross-linking structures in the intracellular formed AGEs, and blocking the receptor for advanced glycation end products (RAGEs) [50]. Further investigations of this plant are required to prove the antiglycation mechanisms described above.

## Conclusions

Eleven aqueous extracts of herbal medicines exhibit the MG-trapping ability and the formation of MG-derived AGEs. *Syzygium aromaticum* and *Phyllanthus amarus* appeared to inhibit the formation of MG-derived AGEs through their MG-trapping abilities. Antiglycation of herbal medicines may be responsible for their usefulness in the management and prevention of AGE-mediated diabetic complication.

## Abbreviations

AGEs: Advanced glycation end-products
BSA: Bovine serum albumin
AG: Aminoguanidine
DPPH: 1,1-diphenyl 2-picrylhydrazyl radical scavenging activity
MG: Methylglyoxal
